# *CSNK1A1* mutations and gene expression analysis in myelodysplastic syndromes with del(5q)

**DOI:** 10.1111/bjh.13563

**Published:** 2015-06-18

**Authors:** Erica Bello, Andrea Pellagatti, Jacqueline Shaw, Cristina Mecucci, Rajko Kušec, Sally Killick, Aristoteles Giagounidis, Sophie Raynaud, María J. Calasanz, Pierre Fenaux, Jacqueline Boultwood

**Affiliations:** ^1^ LLR Molecular Haematology UnitNuffield Division of Clinical Laboratory SciencesRadcliffe Department of MedicineUniversity of OxfordOxfordUK; ^2^ NIHR Biomedical Research CentreOxfordUK; ^3^ Haematology and Bone Marrow Transplantation UnitUniversity of PerugiaPerugiaItaly; ^4^ Dubrava University Hospital and Zagreb School of MedicineUniversity of ZagrebZagrebCroatia; ^5^ Department of HaematologyRoyal Bournemouth HospitalBournemouthUK; ^6^ Department of Haematology, Oncology, and Palliative CareMarienhospital DüsseldorfDüsseldorfGermany; ^7^ Centre Hospitalier Universitaire NiceNiceFrance; ^8^ Department of GeneticsUniversity of NavarraPamplonaSpain; ^9^ Service d'hématologie seniorsHôpital St LouisParisFrance

**Keywords:** CSNK1A1, mutation, haploinsufficiency, 5q‐ syndrome, del(5q)

## Abstract

Mutations of *CSNK1A1*, a gene mapping to the commonly deleted region of the 5q‐ syndrome, have been recently described in patients with del(5q) myelodysplastic syndromes (MDS). Haploinsufficiency of *Csnk1a1* in mice has been shown to result in β‐catenin activation and expansion of haematopoietic stem cells (HSC). We have screened a large cohort of 104 del(5q) MDS patients and have identified mutations of *CSNK1A1* in five cases (approximately 5%). We have shown up‐regulation of β‐catenin target genes in the HSC of patients with del(5q) MDS. Our data further support a central role of CSNK1A1 in the pathogenesis of MDS with del(5q).

The myelodysplastic syndromes (MDS) are a heterogeneous group of clonal haematopoietic stem cell (HSC) malignancies characterized by ineffective haematopoiesis and peripheral blood cytopenias (Heaney & Golde, 1999). MDS patients typically have a hypercellular bone marrow. Approximately 40% of MDS cases progress to acute myeloid leukaemia (Heaney & Golde, 1999).

Deletion of the long arm of chromosome 5 [del(5q)] occurs in approximately 10–20% of patients with *de novo* MDS (Giagounidis *et al*, 2004; Boultwood *et al*, 2010) and is the sole karyotypic abnormality in patients with the 5q‐ syndrome, the most distinct of the MDS (Giagounidis *et al*, 2004; Boultwood *et al*, 2010). The commonly deleted region (CDR) of the 5q‐ syndrome was identified and narrowed to a 1·5 Mb interval at 5q32 (Boultwood *et al*, 2002). Several candidate genes map to the CDR, including *CSNK1A1,* which was found to be haploinsufficient (i.e. down‐regulated by approximately 50%) in the CD34^+^ cells of 5q‐ syndrome patients in a gene expression profiling (GEP) study (Boultwood *et al*, 2007). In this setting the remaining copy of the gene does not compensate the loss of the other allele. Sanger sequencing‐based screening of all 40 genes within the CDR did not identify any mutations in a cohort of ten 5q‐ syndrome patients. However, in a recent study (Schneider *et al*, 2014), mutations of *CSNK1A1* were identified using whole‐exome sequencing in 2 of 19 del(5q) cases and a further *CSNK1A1* mutation was found in an additional cohort of 22 MDS cases with isolated del(5q) using next‐generation targeted sequencing, giving an overall frequency of approximately 7% in the MDS del(5q) cases analysed. This is the first report of mutations in a gene mapping to the CDR of the 5q‐ syndrome, although these mutations are found in a small subset of del(5q) MDS patients (Schneider *et al*, 2014). *CSNK1A1* encodes a serine/threonine kinase (CK1α), which has a regulatory role in the Wnt/β‐catenin and p53 signalling pathways (Elyada *et al*, 2011). Schneider *et al* (2014) showed that expression of mutant *CSNK1A1* resulted in β‐catenin activation and HSC cell cycle progression.

Heterozygous inactivation of *Csnk1a1* in mice also resulted in β‐catenin activation and expansion of HSCs, suggesting that *CSNK1A1* haploinsufficiency may be the mechanism underlying the initial clonal expansion in patients with the 5q‐ syndrome (Schneider *et al*, 2014).

In this study, firstly we have screened a large cohort of MDS cases with del(5q) for mutations in *CSNK1A1*. Secondly, we have investigated the impact of *CSNK1A1* haploinsufficiency and mutation on the expression of β‐catenin‐related genes in the CD34^+^ cells from MDS patients with del(5q) using GEP.

## Materials and methods

### Patient samples

A total of 104 MDS cases with del(5q) were included in this study (Table SI). Genomic DNA was isolated using phenol‐chloroform extraction from bone marrow samples or from peripheral blood neutrophils isolated using Histopaque (Sigma‐Aldrich, Gillingham, UK) and pelleted after hypotonic lysis of erythrocytes.

### Sanger sequencing

Sanger sequencing was performed following polymerase chain reaction (PCR) amplification using the following primers: exon 3 of *CSNK1A1* forward primer 5′‐TCCTTTTGTTTCGTTAGGTGGT‐3′ and reverse primer 5′‐AAGGTTAAATAGTGATGCACAGGA‐3′, exon 4 forward primer 5′‐GCCAAAGGACACAGCAGGTA‐3′ and reverse primer 5′‐CAGCAAATTCAACTTACTATGGC‐3′.

### GEP and data analysis

Gene expression profiling data on CD34^+^ cells from a group of MDS patients with del(5q) and healthy controls were obtained from a dataset previously published by our group (Pellagatti *et al*, 2010). The microarray platform used was the Affymetrix GeneChip Human Genome U133 Plus 2·0 (47 000 transcripts) (Affymetrix, Santa Clara, CA, USA). Analysis of gene set up‐ or down‐regulation was performed using Gene Set Enrichment Analysis (GSEA) as previously described (Papaemmanuil *et al*, 2011).

### Real‐time quantitative PCR

Real‐time quantitative PCR reactions were run on a LightCycler 96 Real‐Time PCR System (Roche Diagnostics, Lewes, UK). Pre‐developed TaqMan Assays were used (Assays‐on‐Demand, Applied Biosystems, Foster City, CA, USA) and the expression level of the beta‐2‐microglobulin gene (*B2M*) was used to normalize for differences in input cDNA. Each sample was performed in triplicate and the expression ratios were calculated using the ΔΔ*C*_t_ method.

## Results and discussion

We have determined the frequency of *CSNK1A1* mutations in a large cohort of 104 cases of MDS with del(5q) using Sanger sequencing. Schneider *et al* (2014) identified *CSNK1A1* mutations in del(5q) MDS in exon 3, and two previous studies (Graubert *et al*, 2012; Woll *et al*, 2014) reported *CSNK1A1* mutations in exon 4 of the gene. We therefore focused our investigation on the analysis of the sequences of exon 3 and 4 of *CSNK1A1*.

**Figure 1:**
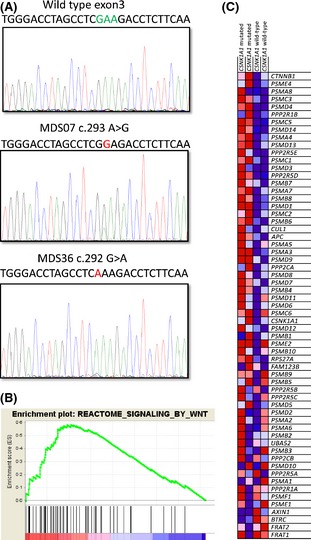
(A) Representative examples of *CSNK1A1* mutations identified in exon 3 of the *CSNK1A1* gene in del(5q) myelodysplastic syndrome (MDS) patients. The top panel shows part of the wild‐type sequence of exon 3 of *CSNK1A1*, with the nucleotides corresponding to Glutamic Acid (Glu) 98 highlighted in green above the relevant sequence peaks. The sequence of two del(5q) MDS patients with *CSNK1A1* mutations in position 98 are shown with the mutation highlighted in red. (B) Enrichment plot of the ‘REACTOME_SIGNALING_BY_WNT’ gene set obtained using Gene Set Enrichment Analysis (GSEA). (C) GSEA‐generated heatmap showing the expression levels (red=high, blue=low) of the genes in the ‘REACTOME_SIGNALING_BY_WNT’ gene set in two del(5q) MDS cases with *CSNK1A1* mutations and two del(5q) MDS cases wild‐type for *CSNK1A1*.

We identified missense mutations of *CSNK1A1* in five del(5q) MDS cases in our cohort (Table 1, Fig 1A). All five patients harbouring *CSNK1A1* mutations had refractory anaemia (two of which had the 5q‐ syndrome). Two of the *CSNK1A1* mutations identified caused a previously reported amino acid change, E98K (Schneider *et al*, 2014). An additional case harboured a different *CSNK1A1* mutation affecting amino acid 98 (E98G), previously described in other malignancies (Dulak *et al*, 2013). Moreover, a previously reported *CSNK1A1* mutation at amino acid 140 (D140A) (Graubert *et al*, 2012) was found in one case of MDS with isolated del(5q) in our study. We identified a novel *CSNK1A1* mutation at codon 134 (H134L) that has not been previously reported. The *CSNK1A1* mutations identified were analysed using the PolyPhen‐2 (http://genetics.bwh.harvard.edu/pph2/) and SIFT (http://sift.jcvi.org/) online tools, in order to predict the effect of the mutations on protein function. All *CSNK1A1* mutations, including the newly identified H134L mutation, were reported as damaging by PolyPhen‐2 and SIFT analysis (Table 1).

**Table 1:** Details of the *CSNK1A1* mutations identified in del(5q) MDS patients and PolyPhen‐2 and SIFT prediction of the effect of the mutations on protein function

Patient ID	Diagnosis	Karyotype	*CSNK1A1* mutation	PolyPhen‐2 prediction/score	SIFT prediction/score
MDS05	RA	46,XX,t(1;3)(p33;p14),del(5)(q14q34)[21]/46,XX[4]	c.401A>T, p.H134L	Probably damaging/1	Damaging/0
MDS07	RA	46,XX,del(5)(q14q34),inv(9)(p11q13)c[30]	c.293A>G, p.E98G	Probably damaging/0·999	Damaging/0
MDS14	RA (5q‐ syndrome)	46,XX,del(5)(q13q33)[26]/46,XX[4]	c.419A>C, p.D140A	Possibly damaging/0·877	Damaging/0
MDS36	RA (5q‐ syndrome)	46,XX,del(5)(q?)[30]	c.292G>A, p.E98K	Probably damaging/0·999	Damaging/0
MDS72	RA	46,XX,del(5)(q?),del(7)(q?)[30]	c.292G>A, p.E98K	Probably damaging/0·999	Damaging/0

MDS, myelodysplastic syndrome; RA, refractory anaemia; SIFT, Sorting Intolerant from Tolerant.

The overall frequency of *CSNK1A1* mutations in our cohort was approximately 5% (5/104 of cases), which is consistent with the previous report suggesting that *CSNK1A1* mutations are rare events in del(5q) MDS (Schneider *et al*, 2014). Patients with del(5q) show haploinsufficiency of *CSNK1A1* (because it maps to the CDR) and a small proportion of these patients also harbour mutation of the remaining allele**.** Next‐generation‐based targeted re‐sequencing data, using a panel targeting 25 genes mutated in various myeloid malignancies (Fernandez‐Mercado *et al*, 2013), were available for four patients with and 37 patients without *CSNK1A1* mutations. The additional mutations found in the cases with *CSNK1A1* mutations were a *RUNX1* mutation in one patient and a *U2AF1* mutation in another patient and we did not therefore observe specific association of *CSNK1A1* mutations with other myeloid gene mutations. However, the number of cases analysed is clearly small, and the study of larger cohorts of del(5q) MDS cases with *CSNK1A1* mutations is required to determine whether robust associations with other gene mutations exist.

It has been recently reported that lenalidomide, a drug widely used to treat del(5q) MDS (List *et al*, 2006), induces the ubiquitination and consequent degradation of *CSNK1A1* by the CRBN‐CRL4 E3 ubiquitin ligase, and that haploinsufficiency of CSNK1A1 might increase lenalidomide sensitivity in del(5q) haematopoietic cells (Fink *et al*, 2014). Knockdown of CSNK1A1 sensitized primary CD34^+^ cells to lenalidomide, suggesting that haploinsufficiency of CSNK1A1 might increase lenalidomide sensitivity in del(5q) haematopoietic cells (Fink *et al*, 2014). One of our cases with the *CSNK1A1* mutation E98K [MDS72, carrying a del(5q) and a del(7q), Table 1] did not respond to treatment with lenalidomide.

*CSNK1A1* encodes a protein that is a major regulator of the Wnt/β‐catenin pathway. We have re‐analysed our existing GEP data on MDS CD34^+^ cells (Pellagatti *et al*, 2010) to determine whether reduced expression of *CSNK1A1* in patients with del(5q) is associated with increased expression of the major downstream effectors of the Wnt/β‐catenin signalling pathway. The average expression fold change (patients *versus* median of 17 healthy controls) for *CSNK1A1* was 0·56 in 16 patients with 5q‐ syndrome and 0·58 in 30 patients with del(5q) (Fig S1), confirming that patients with 5q‐ show haploinsufficient levels of this gene. The average expression fold change for *CCND1* (encoding cyclin D1)*,* a major downstream effector of Wnt/β‐catenin and regulator of cell cycle progression, was 1·42 in patients with 5q‐ syndrome and 1·58 in patients with del(5q) (Fig S1), showing that expression levels of this gene are increased by approximately 50% in patients with 5q‐. These data show that haploinsufficiency of *CSNK1A1* is associated with increased expression of Wnt/β‐catenin downstream effector genes in the HSC of MDS patients with del(5q) and are consistent with the previous demonstration that *Csnk1a1^+/−^* haematopoietic cells transplanted into wild‐type mice showed increased expression of cyclin D1 (accompanied by β‐catenin nuclear accumulation) (Schneider *et al*, 2014).

Gene expression profiling data were available for four patients with del(5q) for which we were able to determine the mutation status of *CSNK1A1*: two patients were mutated and two patients were wild‐type for *CSNK1A1*. We performed GSEA to compare the gene expression profiles of the two patients with *CSNK1A1* mutation with those of the two patients without mutations of this gene, in order to determine whether coordinated up‐regulation of pathways/processes associated with Wnt/β‐catenin function could be observed. The ‘REACTOME_SIGNALING_BY_WNT’ gene set was found to be significantly up‐regulated (*q* < 0·001) in the patients with *CSNK1A1* mutation compared with the patients without *CSNK1A1* mutations (Fig 1B, C). These data suggest that *CSNK1A1* mutations in del(5q) MDS may lead to an increase in the expression of genes involved in Wnt signalling.

In summary, we have confirmed the presence of *CSNK1A1* mutations in a small proportion of patients with del(5q) MDS and shown up‐regulation of β‐catenin target genes in the HSC of patients with del(5q) MDS. Our data support a central role for CSNK1A1 in the pathogenesis of MDS with del(5q).

## Author contributions

AP and JB designed the research study; EB, AP and JS performed the research; CM, RK, SK, AG, SR, MJC, PF and JB contributed patient samples and helped with the analysis of the data; EB, AP, JS and JB analysed the data and wrote the paper.

## Conflicts of interest

The authors have no competing interests.

## Supporting information


**Table SI**. Patient details.

**Fig S1.** Expression ratios for *CSNK1A1* [*n* = 3 cases with del(5q)] and *CCND1* [*n* = 3 cases with del(5q)] obtained from real‐time quantitative PCR (blue bars) and Affymetrix experiments (red bars).
