# Supplementary material for: CSNK1A1 mutations and gene expression analysis in myelodysplastic syndromes with del(5q)
Source: Br J Haematol. 2015 Jun 18;171(2):210–4. doi: 10.1111/bjh.13563 (PMC4744770; doi:10.1111/bjh.13563)
Supplement: Supplementary file 2 — Fig S1. Expression ratios for CSNK1A1 [n = 3 cases with del(5q)] and CCND1 [n = 3 cases with del(5q)] obtained from real‐time quantitative PCR (blue bars) and Affymetrix experiments (red bars). [file BJH-171-210-s002.docx]

**Supplementary Figure 1.** Expression ratios for *CSNK1A1* [n=3 cases with del(5q)] and *CCND1* [n=3 cases with del(5q)] obtained from real-time quantitative PCR (blue bars) and Affymetrix experiments (red bars).
